# Chromatic Contrast Alone Does Not Predict Predation Risk in the Longhorn Beetle *Rosalia alpina*

**DOI:** 10.1002/ece3.73915

**Published:** 2026-06-30

**Authors:** Danilo Russo, Stefano Rambaldi, Luca Cistrone, Antonio Pietro Garonna, Roberta Latini, Maria Buglione, Domenico Fulgione

**Affiliations:** ^1^ Laboratory of Animal Ecology and Evolution (AnEcoEvo), Dipartimento di Agraria Università degli Studi di Napoli Federico II Portici Italy; ^2^ Dipartimento di Agraria Università degli Studi di Napoli Federico II Portici Italy; ^3^ Servizio Scientifico Ente Parco Nazionale d'Abruzzo Lazio e Molise Pescasseroli Italy; ^4^ Dipartimento di Biologia Università degli Studi di Napoli Federico II Naples Italy

**Keywords:** background matching, camouflage, disruptive colouration, *Rosalia alpina*, thermoregulation

## Abstract

Colouration can affect predation risk through camouflage, signalling, or other mechanisms that shape predator perception and decision‐making. The Rosalia longicorn ( *Rosalia alpina* ), a flagship longhorn beetle of European beech forests, has a bluish‐grey exoskeleton with conspicuous black spots, yet the function of this colour pattern remains unclear. We tested whether its blue body colouration is consistent with background matching to beech bark and whether black spotting reduces predation risk beyond that provided by the blue body colouration alone. We measured reflectance spectra of the blue body colouration, black spots and beech bark, modelled chromatic contrasts under avian vision, and deployed plasticine beetle models in three colour variants: black, blue and a natural pattern with blue and black spots. Visual modelling showed that both beetle colours were chromatically distinguishable from beech bark, but black spots had lower chromatic contrast than blue body colouration, suggesting that the black component may match the bark background more closely than the blue component. In the field experiment, attack occurrence varied with colour, sampling session and their interaction. In September, blue and patterned models were less likely than black models to be attacked. Patterned models also received fewer marks than black models. By October, these colour differences had weakened or disappeared. These findings indicate that the blue body colouration and the natural colour pattern may reduce predation risk relative to an entirely black phenotype, particularly early in the season. However, we found no clear evidence that black spotting provides protection beyond that afforded by the blue body colouration alone. Alternative functions of the spots, including thermoregulation or signalling, remain plausible.

## Graphical abstract

Prey colouration can affect predation risk, yet the function of the striking blue‐and‐black pattern of the longhorn beetle *Rosalia alpina* has not been tested experimentally. By combining reflectance measurements, visual modelling of potential avian predators and field experiments with plasticine models, we show that chromatic contrast alone does not predict predation risk and that both colours provide only limited background matching. In September, both the natural pattern and the blue body colouration were associated with reduced attack probability relative to black, with no significant difference between them, whereas only the natural pattern showed a clear reduction in pecking intensity; these advantages disappeared in October. These results indicate that the protective effect of R. alpina colouration is context‐dependent and cannot be explained by chromatic contrast alone, possibly reflecting seasonal changes in predator experience or behaviour.

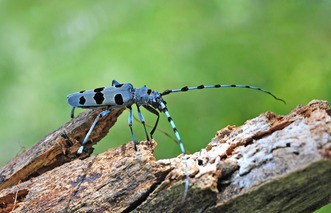

## 1 Introduction

Colouration in animals serves multiple ecological functions, with antipredatory defence among the most widespread and vital (e.g., Caro et al. 2016; Cott 1940; Stevens 2015; Thayer and Thayer 1909). Since Wallace (1867) emphasised the adaptive value of concealment, extensive research has shown that natural selection shapes colour‐based strategies that reduce detectability or deter predators.

Among the most studied mechanisms are background matching and disruptive colouration. Background matching reduces detectability when an organism's body colouration resembles the surrounding substrate, whereas disruptive colouration involves high‐contrast patterns that break up the body outline and hinder recognition (Cuthill 2019; Merilaita et al. 2017; Stevens and Merilaita 2009).

Conspicuous markings, such as spots or patches, may influence these mechanisms depending on their spatial arrangement. When their size, shape, or distribution resembles background elements, they can enhance background matching, whereas high‐contrast markings may be disruptive by fragmenting the body outline, particularly when located near edges (Stevens and Merilaita 2009; Cuthill 2019). At the same time, such markings may also serve non‐cryptic functions, including signalling or deimatic displays (Badejo et al. 2020; Drinkwater et al. 2022), highlighting that their functional role is not necessarily limited to camouflage.

These strategies may act in combination, and their effectiveness depends on environmental context, predator visual systems and behavioural factors.

Importantly, the protective value of colour patterns is not fixed. Predator responses may vary with ecological conditions and prior experience, including the formation of search images (Tinbergen 1960) or reduced neophobia (Crane and Ferrari 2017) toward novel prey types. Consequently, whether conspicuous markings provide additional concealment beyond a well‐matched background, or instead fulfil non‐cryptic functions, remains an open question in many model species. Prey resting on tree bark provides an ideal context for examining these alternatives, as both background matching and high‐contrast patterning may influence detectability under variable lighting and substrate conditions (e.g., de Alcantara Viana et al. 2023).

**FIGURE 1:**
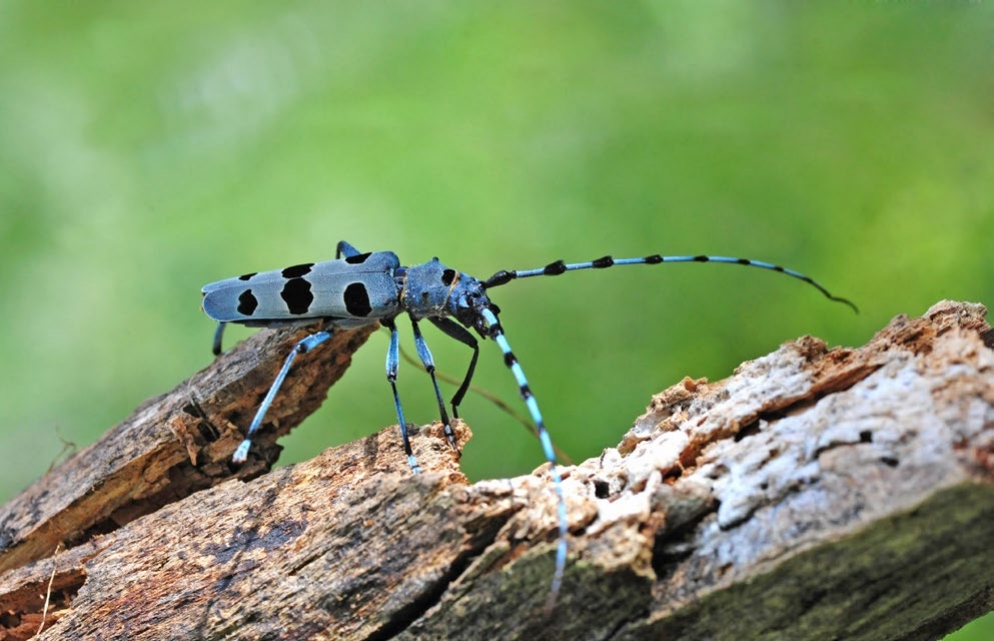
*Rosalia alpina* (L., 1758), showing the characteristic bluish‐grey body colouration and conspicuous black spots on the pronotum and elytra. The species is often found on the trunks of mature or decaying European beech ( *Fagus sylvatica* ) trees in forest habitats. Image courtesy of Angelina Iannarelli.

We investigate how body colouration may reduce predation risk in the Rosalia longicorn ( *Rosalia alpina* Linnaeus, 1758), a large, saproxylic cerambycid beetle easily recognised by its bluish‐grey body (hereafter termed “blue” for simplicity) and conspicuous black spots on the thorax and elytra (Duelli and Wermelinger 2005) (Figure 1). These markings are unique to each individual (Caci et al. 2013).

The beetle's distinctive appearance has contributed to its status as a flagship species for European beech forests (Duelli and Wermelinger 2005). Although historically widespread across southern and central Europe, *R. alpina* has declined due to habitat loss and fragmentation, and the removal of dead or senescent trees (Adamski et al. 2016; Bosso et al. 2013; Campagnaro et al. 2017; Drag et al. 2011; Lachat et al. 2013; Russo et al. 2011). *Rosalia alpina* develops in decaying broadleaved wood and shows a strong preference for European beech, *Fagus sylvatica* (Russo et al. 2011, 2015), with which it shares a closely aligned phylogeographic history (Drag et al. 2018), and today persists mainly in mature beech forests characterised by abundant coarse woody debris (e.g., Campanaro et al. 2017; Castro and Fernández 2016; Drag et al. 2011, 2018; Duelli and Wermelinger 2005; Russo et al. 2011, 2015).

The life cycle of *R. alpina* lasts at least three years, with larvae developing inside decaying wood (Drag et al. 2011). Adults emerge in summer and are active for only a few weeks. During this period, females often remain stationary on the trunks of dead or dying trees while waiting for mates, whereas males patrol the bark surface in search of receptive partners (Bense et al. 2003; Drag et al. 2011; Duelli and Wermelinger 2005). This slow and largely sedentary behaviour, combined with diurnal activity, likely increases exposure to visually hunting predators, such as birds, which prey on the species (Adamski et al. 2013; Castro et al. 2019).

Despite its strikingly contrasting colour pattern, *R. alpina* is often assumed to rely on camouflage (Kostić et al. 2016; Pavlović et al. 2018; Starzyk 2004), yet this assumption has never been experimentally tested. The typical blue body colouration may resemble sunlit beech bark and could facilitate background matching, while the black spots may influence detectability by altering local contrast and edge structure.

At the same time, the micro‐ and nanostructured setae covering the elytra enhance light absorption in the visible spectrum and thermal emission in the infrared, contributing to thermal balance under intense solar radiation (Kostić et al. 2016; Pavlović et al. 2018). These findings suggest that the colouration of *R. alpina* may be multifunctional, potentially contributing to thermoregulation and predator avoidance, with its antipredatory value depending on ecological context and predator perception.

In this study, we assess whether the colour pattern of *R. alpina* reduces predation risk and, if so, which components contribute to any protective effect. Specifically, we tested whether the blue colouration is consistent with background matching to beech bark and whether black spots provide an additional reduction in predation risk beyond that associated with the blue colouration alone. Our experimental design tests for an additional antipredatory effect of spots, but does not allow us to distinguish among the specific visual mechanisms that may underlie it.

To evaluate these predictions, we first compared the spectral properties of beetle colours and beech bark under avian vision. We then conducted a field experiment deploying plasticine models in three colour treatments (black, blue and blue with black spots) to quantify predator marking under natural conditions.

If blue colouration enhances camouflage through background matching, the blue body colouration is predicted to show low chromatic contrast against beech bark, and both blue and naturally patterned models would experience lower predation than uniformly black models. Alternatively, if black spots confer an added antipredatory benefit, models bearing the natural colour pattern should be less frequently attacked than both uniformly blue and black models. We treat this as a general “added‐benefit‐of‐spots” prediction rather than a definitive test of disruptive effect per se.

## 2 Materials and Methods

### 2.1 Reflectance Analysis

**FIGURE 2:**
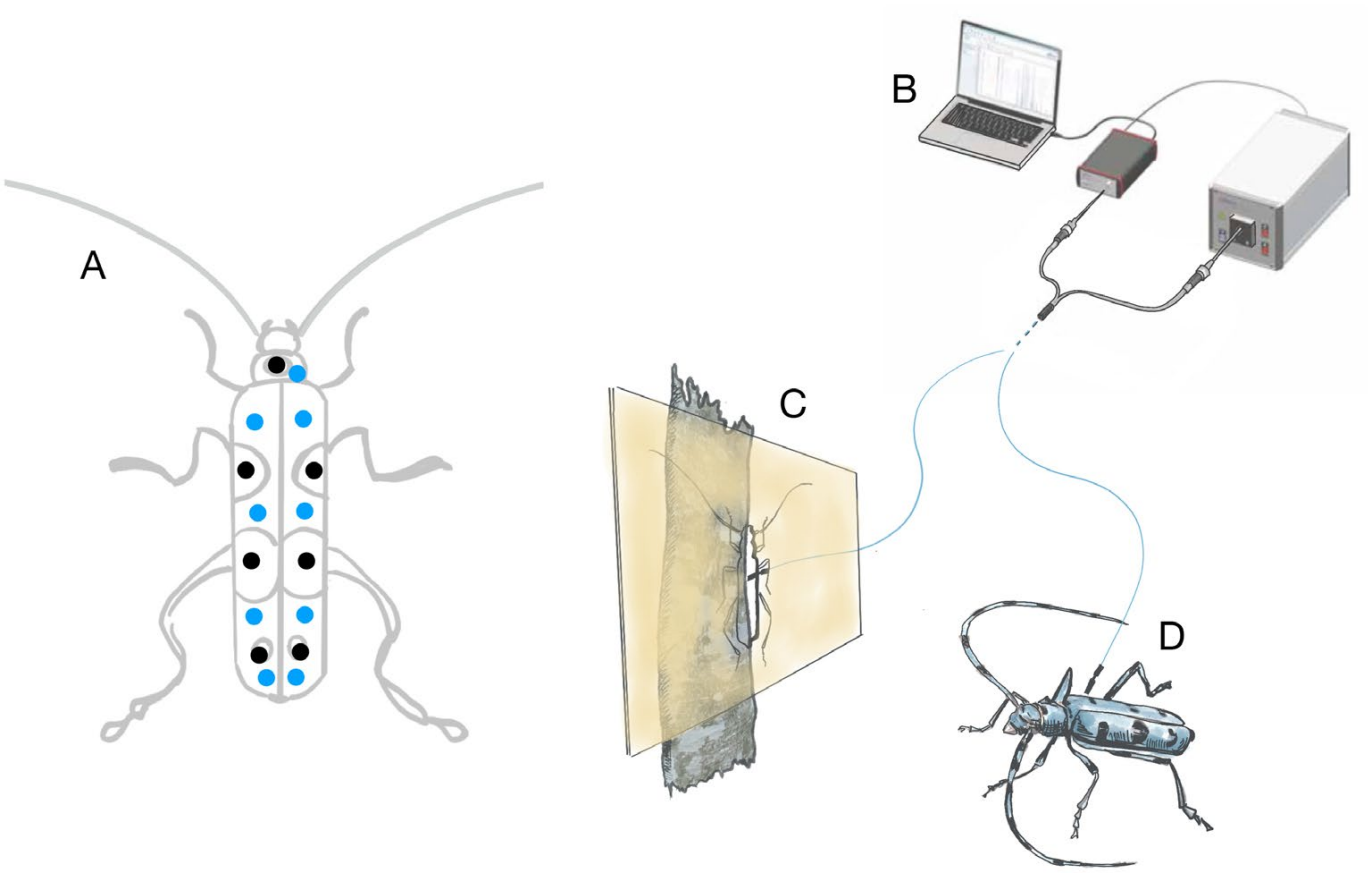
Reflectance measurement setup for *Rosalia alpina* . (A) Measurement points on blue (light blue dots) and black (black dots) areas of the pronotum and elytra. (B) Spectrophotometer setup (AvaSpec‐2048‐USB2‐UA‐50, 250–1000 nm) connected to a computer, a light source (AvaLight‐DH‐S‐BAL light source) and a standard fibre‐optic reflection probe. (C) Measurement of wood fragments bearing beetle silhouettes. (D) Example of reflectance measurement on a beetle, with the probe (0.2 mm hole end) positioned perpendicular to the surface to record reflectance (R%).

To characterise the reflectance of *R. alpina* colouration, we analysed 30 adult specimens (28 from the University of Naples Federico II's MUSA Museum, Portici (Italy) and two found dead at the Abruzzo, Lazio and Molise National Park, hereafter PNALM). The use of preserved individuals was necessary because *R. alpina* is a strictly protected species under the EU Habitats Directive, which limits the collection and manipulation of live individuals for laboratory analyses. Only specimens showing no tangible signs of physical deterioration were included in the analysis. Spectral measurements were taken using an AvaSpec‐2048‐USB2‐UA‐50, 250–1000 nm spectrophotometer (Avantes, Apeldoorn, Netherlands) coupled with an AvaLight‐DH‐S‐BAL light source and a standard fibre‐optic reflection probe. In this configuration, illumination and collection are integrated into the probe at a fixed, manufacturer‐defined geometry, ensuring constant and reproducible optical conditions across all measurements. As such, the illumination geometry is fixed by the probe design and does not allow independent adjustment or specification of a light‐source angle. The spectrophotometer probe (with a 0.2 mm‐hole end) was positioned perpendicular to the animals' body surface to maintain a standardised measurement setup and was used to record reflectance (R%) (Fulgione et al. 2019). The instrument was calibrated at the start of each measurement session using absolute white and black calibration standards (WS2; Avantes). For each specimen, we measured nine blue areas (one on the pronotum and four on each elytron, all located between black spots) and seven black spots (one on the pronotum and six on the elytra), yielding nine blue and seven black reflectance curves per individual (Figure 2; File S1: Tables S1 and S2).

We also analysed 30 fragments of beech wood collected from trees used by *R. alpina* for reproduction in the PNALM area (Russo et al. 2011), representing different stages of decay. Trees were deemed suitable based on the presence of characteristic larval galleries and emergence holes of *R. alpina* . Wood fragments were collected from multiple trees to capture naturally occurring variability in bark condition and decomposition stage, rather than standardising substrate characteristics.

On each fragment, we positioned a life‐sized cut‐out of the beetle's dorsal silhouette, derived from museum specimens. Within each silhouette, eight measurement points (four per side) were recorded to characterise the background reflectance experienced by a resting beetle. Silhouettes were placed at 5 cm intervals along each fragment, with the number of placements (1–6) depending on fragment length (Figure 2). This approach ensured that reflectance comparisons between beetles and substrate were made over spatially corresponding areas, accounting for the high heterogeneity of bark surfaces and avoiding mismatches between colour measurements and background context.

All fragments and corresponding measurement sets were assigned unique codes and photographed for documentation. For each specimen or wood fragment, we calculated the average reflectance across the blue, black and substrate measurements.

### 2.2 Reflectance Data Processing and Perceptual Analysis

All reflectance spectra were exported as matrices and converted to CSV files. For each beetle specimen, reflectance spectra were averaged across measurement points to obtain one mean blue and one mean black spectrum per individual. For each wood fragment, spectra were averaged across measurement points and silhouette placements, resulting in one mean substrate spectrum per fragment.

Analyses were conducted in RStudio using the *pavo* package (version 2.10.0), which processes spectral data according to different animal visual systems (Maia et al. 2013, 2019). For our goals, spectral analysis was limited to wavelengths between 300 and 700 nm, corresponding to the visual range of most birds (Maia et al. 2013, 2019; Vorobyev and Osorio 1998). CSV files were imported as rspec objects and converted to perceptual models (vismodel) using standard parameters for avian tetrachromatic vision (UV, S, M, L cones) and applying a standard D65 irradiance spectrum.

We estimated chromatic contrasts (ΔS), expressed in units of Just Noticeable Differences (JNDs), between beetle colour and the wood substrate using the coldist function in *pavo*, which implements the receptor‐noise limited model of Vorobyev and Osorio (1998) with a Weber fraction *ω* = 0.1, appropriate for avian vision. For each individual beetle, ΔS values were calculated for blue versus substrate and black versus substrate. Differences in chromatic contrast between blue body colouration and black spots were assessed using Wilcoxon signed‐rank tests because the data did not meet the assumptions of normality.

### 2.3 Predation Experiment

**FIGURE 3:**
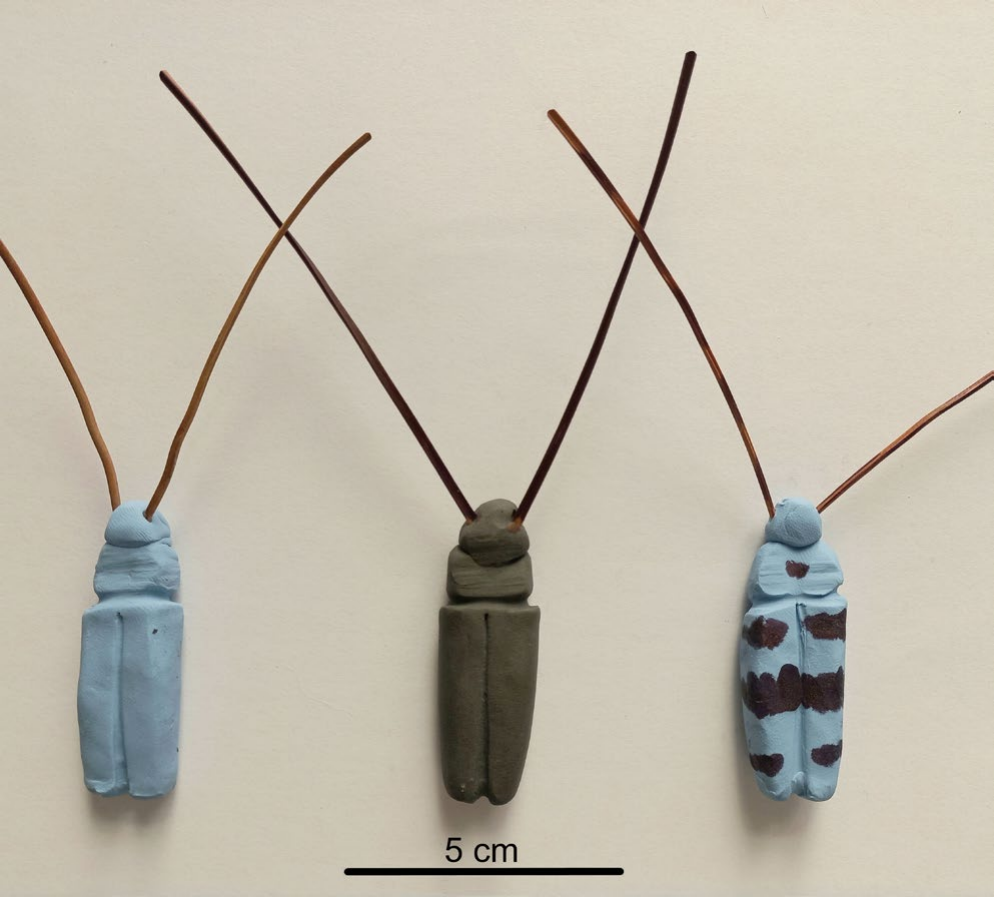
Plasticine models of *Rosalia alpina* used in the predation experiment. Three colour variants were prepared: Blue (matching the beetle's background colour), black and natural‐patterned (blue with back spots).

To estimate predation pressure and assess the effect of colouration on potential attacks, we used artificial models made from commercial non‐toxic plasticine. This method isolates colour as the only salient trait detectable by predators, removing confounding cues such as movement or scent. Models were hand‐made in three colour variants: entirely black, entirely blue (matching the beetle's background colour), and blue with black spots reproducing the *R. alpina* pattern (Figure 3). Each model measured approximately 3 cm in length, < 2 cm in width and about 5 mm in thickness, with a flattened cylindrical body and a spherical head (approximately 1 cm in diameter). A small wooden toothpick served as internal support for mounting, and two pine needles were inserted to mimic antennae. For patterned models, black spots were manually drawn using a permanent, biodegradable black marker. Each colour variant was represented by 80 models, for a total of 240. Of these, 239 were recovered and scored for predation marks.

Because the blue plasticine appeared more saturated to the human eye than the natural bluish grey of *R. alpina* , we conducted a spectral validation to quantify perceptual similarity between artificial models and real beetles under avian vision. We measured 12 blue and four black artificial models using the same spectrophotometric setup described above. These spectra were compared to those obtained from 12 blue areas and four black spots measured on randomly selected beetles (File S1: Table S3). Chromatic contrasts between artificial models and real beetle colours were calculated using the same perceptual modelling procedure described for the insect–substrate comparison to confirm perceptual consistency.

The experiment was conducted in a beech forest near Pescasseroli (L'Aquila, Italy; 41.8083° N, 13.7890° E) within the PNALM, where a well‐studied *R. alpina* population occurs (Russo et al. 2011, 2015). Two experimental sessions were conducted, each lasting eight days: the first in the latter half of September and the second in the second week of October. These periods encompassed the late phase and the immediate post‐peak phase of adult activity (July–September), thereby reducing the likelihood that interactions between live beetles and artificial models would influence predator responses and ensuring that predator behaviour was directed exclusively toward the experimental stimuli.

In each session, 120 models were deployed (40 per colour variant), each on a different beech tree considered suitable for *R. alpina* based on previous studies (Russo et al. 2011, 2015) and the presence of larval galleries. Tree assignment, model colour, model height above ground (1.5–3 m) and placement aspect on the trunk were fully randomised. Inter‐tree distance was not standardised because the design aimed to randomise model placement among suitable trees within the study area. Because each tree hosted a single model and trees were not reused, tree identity could not be included as a random effect, as no replication was available at that level. Instead, tree‐related variability was addressed by fully randomising model placement across trees. Models were attached to the right elytron using small metal nails.

During each session, models were inspected twice over the 8‐day trial to ensure that they were still in place, and the total number of marks accumulated over the entire trial was counted at the end of each trial and used in the analyses. Predation marks were identifiable as impressions in the plasticine and were photographed. For each model, we recorded whether at least one mark was present (attack occurrence: yes/no) and the total number of marks accumulated during the trial. All visible marks were counted without distinguishing predator type (e.g., birds or mammals), and multiple marks on the same model were summed. Because multiple marks on a single model are not independent once a model has been discovered, our primary response variable was “attack occurrence” (attacked: yes/no), while “total mark counts” were analysed as a secondary measure of pecking intensity. Attack occurrence indicates at least one detection, whereas total mark counts provide additional information on predators' interaction intensity following detection.

At the end of each session, all models were removed. Marks were counted in the field and subsequently verified under a Leica Microsystems EZ 4 stereoscope in the laboratory. The effect of colouration on predation was analysed in Jamovi (v. 2.6.44) using: (a) a binomial logistic regression for attack occurrence (attacked: yes/no), with model colour, session and their interaction as fixed effects; and (b) a negative binomial GLM (log link) for total mark counts (“pecking intensity”), including model colour, session and their interaction as fixed effects. Post hoc comparisons for the colour × session interaction were conducted using Bonferroni‐corrected pairwise comparisons among colour variants. Because each tree hosted a single model and trees were unique across sessions, tree identity was not included as a random effect because no replication was available at the tree level. Statistical significance was set at *p* = 0.05.

## 3 Results

### 3.1 Reflectance Analysis

**FIGURE 4:**
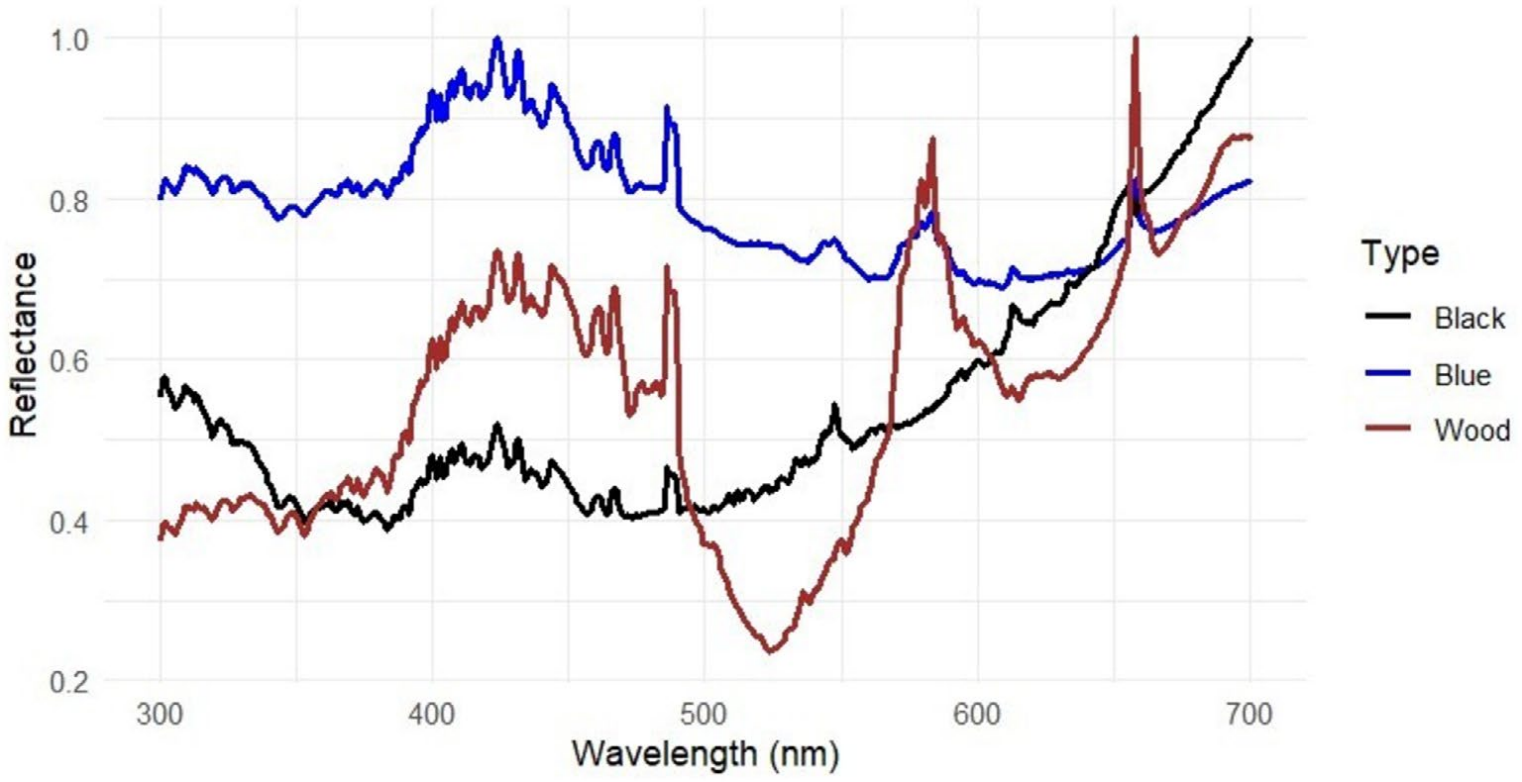
Normalised reflectance spectra of *Rosalia alpina* and its beech ( *Fagus sylvatica* ) substrate. Curves show mean reflectance spectra (300–700 nm) for blue body colouration and black spots of the beetle's exoskeleton (*n* = 30 specimens) and for beech bark (*n* = 30 fragments). For visual comparison of spectral shape, each spectrum was normalised by dividing its reflectance values at each wavelength by the maximum reflectance of that spectrum, prior to averaging. Consequently, the curves depict the relative spectral shape rather than the absolute reflectance magnitude. Perceptual analyses (ΔS) were conducted on non‐normalised reflectance spectra.

Across both beetles' colour components (blue background and black spots) and beech bark, spectra exhibited broadly similar shapes, with peaks around 430, 445, 448, 460, 467, 487 and 660 nm (Figure 4). In chromatic contrast analyses, both the blue body colouration and the black spots of *R. alpina* showed moderate chromatic contrast against beech bark. Chromatic contrast relative to the bark was significantly higher for the blue body colouration (mean ΔS = 4.50 JND, median = 4.25, SD = 1.75) than for the black spots (mean ΔS = 3.04 JND, median = 2.86, SD = 1.04). This difference in chromatic contrast relative to the bark was statistically significant (Wilcoxon signed‐rank test, *W* = 392, *p* < 0.001).

### 3.2 Predation Experiment

Chromatic contrast analyses confirmed that the colours of the plasticine models closely matched those of real beetles. For blue, the mean chromatic distance between models and beetles was Δ*S* = 0.84 JND (median = 0.78, range = 0.53–1.60), with most values below the commonly accepted JND = 1 discrimination threshold. Similarly, black models showed low chromatic contrast relative to natural black spots (mean Δ*S* = 0.31 JND, median = 0.24, range = 0.03–0.83), also below this threshold. These results indicate that artificial models approximated the corresponding beetle colours within the limits of avian chromatic discrimination.

**FIGURE 5:**
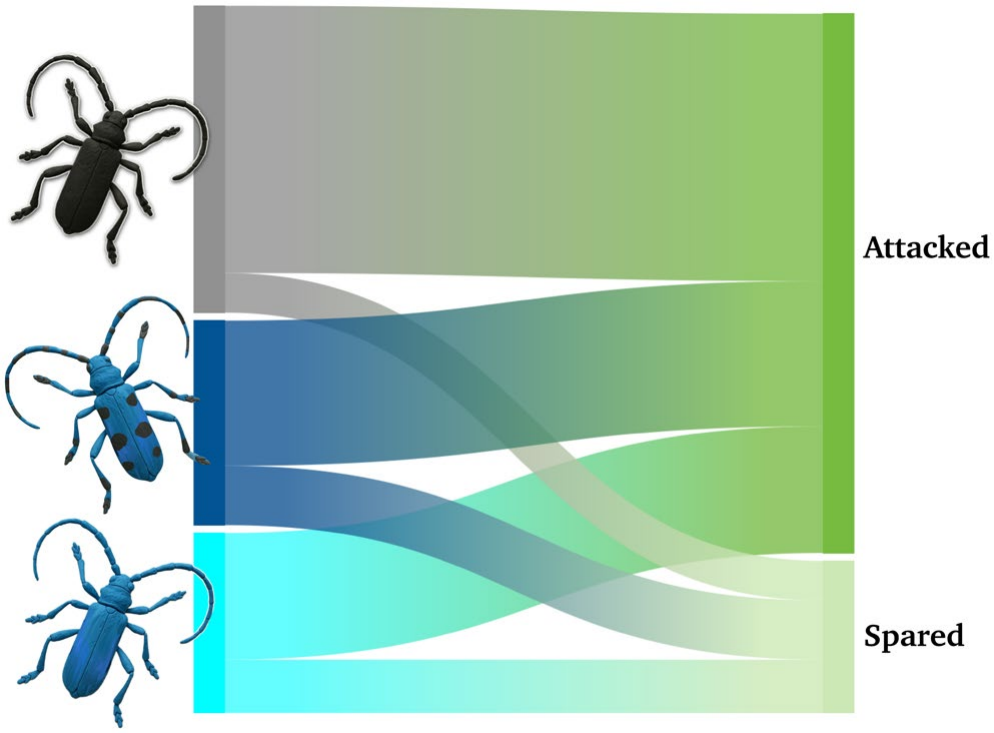
Predation outcomes of *Rosalia alpina* plasticine models. Sankey diagram showing the proportion of black, blue and natural patterned models (blue with black spots) that were either attacked or spared by predators during field experiments in beech forests of the Abruzzo, Lazio and Molise National Park (*n* = 239). Models were classified as attacked if ≥ 1 mark was present. Statistical results from the binomial logistic regression are provided in Table 1.

Of 240 models deployed, 239 were recovered and included in the analysis (File S1: Table S4). One model was lost, possibly removed by a predator or detached from the tree. The binomial logistic regression model including colour, session and their interaction was statistically significant (*n* = 239, AIC = 317, *χ* ^2^ = 24.3, df = 5, *p* < 0.001; Table 1). Figure 5 provides a visual summary of observed attack outcomes, while statistical results from the binomial logistic regression are reported in Table 1.

**TABLE 1:** Model coefficients for binomial logistic regression of attack occurrence (attacked: yes/no), with model colour, session and their interaction as fixed effects.

Predictor	Estimate	SE	*Z*	*p*
Intercept	0.201	0.318	0.631	0.528
Colour				
Patterned vs. black	−1.751	0.524	−3.345	< 0.001
Blue vs. black	−1.012	0.471	−2.150	0.032
Session				
October versus September	0.162	0.455	0.357	0.721
Colour × session				
(Patterned vs. black) × (October vs. September)	1.589	0.694	2.291	0.022
(Blue vs. black) × (October vs. September)	0.795	0.652	1.219	0.223

*Note:* The model estimates the log‐odds of being attacked. Black models and September were used as reference levels; therefore, the main colour effects refer to September. Negative coefficients indicate a lower probability of attack relative to the reference category.

In September, both patterned and blue models had a significantly lower probability of being attacked than black models (patterned vs. black: *p* < 0.001; blue vs. black: *p* = 0.032; Table 1). The patterned and blue models did not differ significantly (*p* = 0.172). The positive interaction between patterned models and October indicates that the difference between patterned and black models weakened in October (*p* = 0.022; Table 1). No significant interaction was detected between blue models and October (*p* = 0.223; Table 1).

**FIGURE 6:**
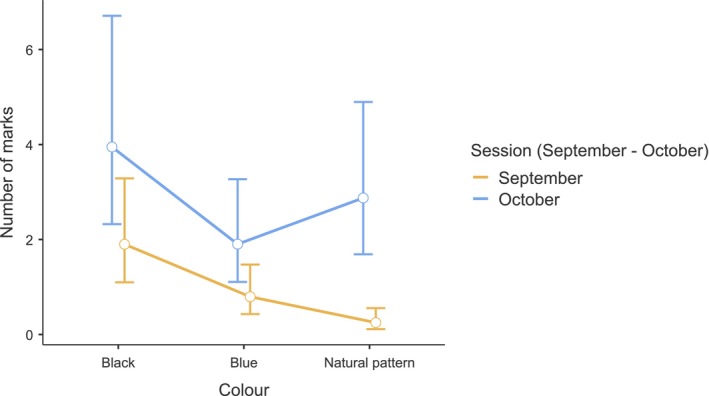
Pecking intensity on *Rosalia alpina* plasticine models across colour treatments and sampling sessions. Model‐predicted expected number of attack marks (estimated marginal means ±95% confidence intervals) from a negative binomial GLM fitted to total mark counts on models deployed on beech trunks in Abruzzo, Lazio and Molise National Park. Lines connect colour treatments within each session (September and October), illustrating the colour × session interaction in mark accumulation (*n* = 239).

For pecking intensity (total number of marks), a negative binomial GLM revealed significant effects of colour (*χ* ^2^ = 16.84, df = 2, *p* < 0.001) and session (*χ* ^2^ = 29.13, df = 1, *p* < 0.001), as well as a significant colour × session interaction (*χ* ^2^ = 8.81, df = 2, *p* = 0.012; Figure 6). The full model fit the data better than the intercept‐only model (AIC full = 792.42; AIC null = 820.35).

Post hoc comparisons (Bonferroni‐corrected) indicated that in September, patterned models received significantly fewer marks than black models, whereas blue models did not differ significantly from either treatment. In October, no significant differences among colour variants were detected. The interaction was primarily driven by a marked increase in pecking intensity on patterned models from September to October (Figure 6).

## 4 Discussion

### 4.1 Chromatic Contrast Is an Incomplete Predictor of Predation

Previous studies, without direct experimental testing, suggested that the colouration of *R. alpina* plays an antipredatory role (Kostić et al. 2016; Pavlović et al. 2018; Starzyk 2004). Our results provide the first experimental evaluation of this assumption and reveal a more nuanced picture than simple background matching. Although chromatic modelling indicated that black spots were more similar to beech bark than the underlying blue colouration, black models were more vulnerable to attack, particularly in September. In the attack‐occurrence analysis, both blue and naturally patterned models had lower attack probability than black models in September, whereas the difference between patterned and black models weakened in October. Similarly, patterned models showed reduced pecking intensity relative to black models in September, but this difference disappeared in October.

This mismatch between chromatic similarity (Δ*S* values) and predation outcomes indicates that colour‐based protection in *R. alpina* cannot be explained solely by static measures of chromatic contrast under controlled conditions. The receptor‐noise limited model (Vorobyev and Osorio 1998) estimates chromatic discriminability under standardised illumination, yet natural forest environments involve variable light regimes, shadowing, bark heterogeneity and complex three‐dimensional surface structure.

Although we focused on chromatic contrast in the visual modelling, achromatic (luminance) contrast, edge detection and surface properties may contribute substantially to detectability (Stevens 2015; Cuthill 2019), particularly under natural lighting conditions. Thus, lower chromatic contrast does not necessarily translate into reduced perceptual salience in the field. We did not explicitly quantify achromatic JNDs, as our modelling framework was designed to test chromatic differences. Moreover, the field predation experiment inherently integrated all perceptual cues available to predators, including luminance, thereby providing a biologically relevant assessment of overall detectability.

In our reflectance analysis, most specimens were drawn from a museum collection. Structural colours, particularly in the blue range, may be sensitive to preservation and storage conditions, potentially affecting absolute reflectance values. However, any such effects are likely to be modest relative to the natural variation in colouration observed among individuals in wild populations. In addition, our conclusions do not rely solely on spectrophotometric data but are supported by the field predation experiment using artificial models, which provides an independent line of evidence unaffected by preservation artefacts.

A further source of variation in our reflectance measurements is the natural heterogeneity of the beech bark substrate. Camouflage through background matching is not solely a function of colour similarity but also depends on the background's complexity and heterogeneity (de Alcantara Viana et al. 2022; Murali et al. 2021). The bark of decaying beech trunks varies visually, with differences in decay stage, fungal colonisation and exposure. Such heterogeneity could modulate the effectiveness of crypsis, potentially favouring colour traits that provide adequate concealment across multiple microhabitats rather than a perfect match to a single substrate. Rather than standardising the decomposition stage or bark characteristics, we deliberately sampled wood fragments from multiple trees used by *R. alpina* to capture the full range of conditions the species encounters in the field. While this variability may increase noise in spectral comparisons, it also offers a more ecologically realistic representation of the visual background against which beetles are detected. Accordingly, substrate heterogeneity should be treated as part of the ecological context rather than as a confounding factor.

Our analysis was also limited to the 300–700 spectrum, including both the ultraviolet and visible components relevant to avian tetrachromatic vision. Wavelengths beyond this range were excluded because they are not expected to contribute to visual perception in the predator model considered; however, future studies might investigate this aspect.

In our predation experiment, the blue body colouration, despite showing moderate chromatic contrast relative to bark, was associated with reduced vulnerability compared to uniformly black models, especially early in the experiment. The effectiveness of background matching, however, also depends strongly on prey density and environmental heterogeneity (Medina et al. 2025). *Rosalia alpina* occurs at low densities even in favourable sites (Russo et al. 2011; Drag et al. 2011), and adults are often observed resting on partially shaded trunks (Castro et al. 2019), conditions that may interact with colouration to reduce detectability.

Microhabitat variability among trees may also have contributed to unexplained variance, yet this is an inherent feature of field experiments and was mitigated by randomisation. Furthermore, behavioural adjustments, such as microhabitat choice, body orientation, or movement suppression, which are absent in static models, could influence the protective function of colour patterns. In moths, for example, selecting suitable backgrounds and adopting specific body orientations are key behaviours enhancing camouflage (Kang et al. 2015), and behavioural adjustments may respond to the degree of background matching (Polverino et al. 2022). Such behavioural plasticity, inevitably excluded in model‐based experiments, may interact with colouration to shape predation risk.

Our experimental design comprised 80 models per colour treatment, distributed across two sampling sessions. We did not conduct an a priori power analysis; sample size was determined by the logistical constraints of the field experiment and by the aim of maintaining balanced replication across colour treatments and sampling sessions. Field experiments inevitably involve residual environmental variability, but the achieved replication and the randomised design support the robustness of the observed main patterns.

We did not classify attacks by predator type because the marks left on the models were not consistently diagnostic enough to allow reliable attribution. We therefore adopted a conservative approach by pooling all attacks, favouring robustness over detail. This choice may reduce the direct correspondence between our avian visual modelling and the full set of recorded predation events, because mammals, reptiles and other predators may differ from birds in their visual systems and foraging strategies. However, birds are widely recognised as the primary visually oriented predators of diurnal, bark‐dwelling insects and are therefore the most likely agents of predatory pressure driving colour selection. Nevertheless, predation pressure on *R. alpina* remains poorly documented, and available information on its natural enemies is largely anecdotal. Future work should identify predator groups to determine whether the protective function of colour patterns in this species is predator‐specific.

The high vulnerability of uniformly black models indicates that dark colouration alone does not confer effective concealment on beech bark, despite its lower chromatic contrast relative to the substrate. In the same forest, the syntopic longhorn beetle *Morimus asper* is uniformly dark and has been observed to rely on alternative defensive behaviours, such as stridulation and the release of defensive secretions when disturbed (pers. obs.). The strong predation pressure observed on black models in our experiment suggests that dark bark colouration may entail substantial risk unless complemented by additional defensive mechanisms. This comparison further illustrates that visually similar substrate use does not necessarily imply equivalent antipredatory strategies.

### 4.2 Temporal Dynamics, Predator Experience and the Role of Black Spots

A second key finding of our study is the strong temporal component of predation risk. In the attack‐occurrence analysis, the difference between patterned and black models weakened from September to October, as shown by the colour × session interaction. A similar temporal pattern emerged for pecking intensity: patterned models received fewer marks than black models in September, but this difference disappeared in October, when attack intensity increased across treatments, including on patterned models. Therefore, any protective advantage associated with the natural colour pattern was transient rather than fixed.

One plausible explanation is that predator responses changed through learning. Search‐image formation, originally defined as a perceptual process of selective attention (Tinbergen 1960), can bias predators toward features of the prey type most frequently encountered during recent searching, increasing detection of the attended type while alternative types are overlooked (e.g., Dawkins 1971; Dukas 2002; Ishii and Shimada 2010). Such biased searching provides a mechanistic basis for frequency‐dependent predation, whereby prey types that are rare or unfamiliar may initially escape focused attention but become increasingly targeted as predators gain experience.

In our case, this framework is consistent with the marked increase in attacks later in the season, particularly on the naturally patterned models. Uniformly dark stimuli may also correspond to a more commonly encountered visual category on tree trunks, including other bark‐dwelling beetles. If predators develop search images biased toward frequently encountered dark silhouettes, uniformly black models may be detected more readily, whereas less common colour configurations could initially escape focused attention.

Reduced neophobia may provide a complementary explanation. Naïve predators can initially avoid novel or uncommon stimuli, but this avoidance may weaken with repeated exposure (Crane and Ferrari 2017). Given the short and seasonal appearance of *R. alpina* adults, predators, especially young individuals, may have limited prior experience with similarly coloured prey early in the season. Over time, improved selective attention (search image) and reduced hesitation toward unfamiliar stimuli (neophobia) could jointly contribute to the observed seasonal reduction of any colour‐associated advantage.

Although we cannot disentangle these mechanisms, the temporal changes observed in both attack occurrence and pecking intensity suggest that predator experience and/or predator community turnover may play an important role in shaping temporal variation in attack patterns.

The attack‐occurrence analysis provided no evidence that naturally patterned models were less vulnerable than blue models, indicating no clear protective advantage of black spots beyond the blue body colouration under our field conditions. Likewise, in the pecking‐intensity analysis, patterned models showed reduced marking relative to black models only early in the season but converged with the other treatments later in the season. These findings do not provide evidence that black spots consistently enhance protection beyond that associated with the blue body colouration alone. In addition to potential effects on detectability, the black spots may also fulfil non‐cryptic functions, as suggested in previous studies, for example, in social interactions (Badejo et al. 2020) and in thermoregulation (Kostić et al. 2016; Pavlović et al. 2018).

Although the spotted pattern may alter local contrast or visual structure, our experimental design did not allow us to directly assess specific mechanisms. Recent quantitative approaches, such as the Quantitative Colour Pattern Analysis (QCPA) framework, could be used in future studies to investigate whether the black spots affect detectability through specific spatial properties of the colour pattern (van den Berg et al. 2020).

Under the conditions tested, the blue body colouration appears sufficient to account for the reduced vulnerability of the natural phenotype relative to uniformly black models, while any additional contribution from spotting, if present, is likely to be context‐dependent.

The distinction between attack occurrence and pecking intensity offers further insight into predator–prey dynamics. The binomial model reveals whether a model was detected and attacked at least once, whereas the negative binomial model quantifies the degree of predator interaction after detection. Both analyses indicate that the protective effect of colouration was strongest early in the experiment and weakened later, particularly for naturally patterned models. However, the two response variables capture different components of predation risk: attack occurrence reflects initial detection or approach decisions, whereas pecking intensity reflects predator behaviour after detection. The increase in pecking intensity on patterned models in October, therefore, reflects a loss of the initial protective advantage observed in September, rather than a reversal of the pattern. These results indicate that detection and post‐detection interaction may both vary over time, potentially due to learning, reduced neophobia, or shifts in the predator assemblage.

By conducting the experiment outside the beetle's peak activity period, we ensured that predator responses were directed solely toward the experimental stimuli. However, predator behaviour may vary seasonally, and predation pressure or selectivity could differ during the period when adult beetles are naturally present. As a result, our estimates may not fully reflect the absolute magnitude of predation risk or its variation among colour patterns during the peak activity period. Nevertheless, the comparative differences among colour treatments remain informative, as all models were exposed under the same environmental conditions. Our results should therefore be interpreted primarily in terms of relative, rather than absolute, differences in predation risk.

## 5 Conclusions

We remark that our inference is geographically limited: experiments were conducted in a single beech forest landscape with avian visual modelling. The relative value of colour traits could differ across alternative backgrounds, light regimes, predator communities, or prey frequencies, consistent with context‐dependent selection on antipredator colouration (Medina et al. 2025).

Collectively, our findings show that colouration in *R. alpina* influences predation risk, but not in a manner fully predicted by chromatic similarity alone. The blue body colouration was associated with reduced attack probability compared with uniformly black colouration, especially early in the experiment, whereas black spots did not confer clear additional protection beyond the blue body colouration under the tested conditions. Importantly, the protective value of the natural phenotype appears temporally dynamic, diminishing as the season progresses. This pattern is consistent with predator learning, search‐image formation, or reduced neophobia, underscoring that antipredator colouration should be interpreted within an ecological and behavioural framework rather than solely through static perceptual metrics.

A potentially important conservation implication emerges when our evidence that colour‐mediated protection is not absolute is considered alongside previous research on the thermal ecology of *R. alpina*. Sun‐exposed trunks provide favourable conditions for egg and larval development and are therefore used by reproductive adults (Russo et al. 2011, 2015), yet canopy openness may increase exposure to predators (Castro et al. 2019; Goßmann et al. 2023). This pattern is consistent with a potential trade‐off between thermal benefits and predation risk, although this mechanism was not directly tested in the present study. Maintaining structurally heterogeneous forests, with a mosaic of shaded and sunlit trunks and varying canopy closure, may help balance these opposing selective pressures and support both reproductive success and survival.

## Author Contributions

**Danilo Russo:** conceptualization (lead), data curation (lead), formal analysis (lead), investigation (lead), methodology (equal), project administration (lead), resources (lead), software (lead), writing – original draft (lead), writing – review and editing (lead). **Stefano Rambaldi:** data curation (equal), formal analysis (lead), investigation (equal), methodology (equal), validation (equal), writing – original draft (supporting), writing – review and editing (supporting). **Luca Cistrone:** data curation (supporting), resources (supporting), validation (equal), writing – original draft (supporting), writing – review and editing (supporting). **Antonio Pietro Garonna:** data curation (supporting), resources (supporting), validation (equal), writing – original draft (supporting), writing – review and editing (supporting). **Roberta Latini:** data curation (supporting), resources (supporting), validation (equal), writing – original draft (supporting), writing – review and editing (supporting). **Maria Buglione:** conceptualization (lead), data curation (equal), formal analysis (equal), investigation (lead), methodology (lead), validation (equal), visualization (equal), writing – review and editing (equal). **Domenico Fulgione:** conceptualization (lead), data curation (equal), formal analysis (lead), funding acquisition (lead), investigation (lead), methodology (equal), project administration (lead), resources (equal), software (equal), writing – review and editing (equal).

## Funding

This study was funded by the Parco Nazionale d'Abruzzo Lazio and Molise.

## Conflicts of Interest

The authors declare no conflicts of interest.

## Supporting information


**Table S1:** Mean reflectance values between approximately 174 and 1100 nm for the blue body colouration of 30 *Rosalia alpina* specimens (ra_01‐ra_30). Each column represents the mean reflectance curve for one individual, calculated from nine blue areas (one on the pronotum and four on each elytron, all located between black spots).
**Table S2:** Mean reflectance values between approximately 174 and 1100 nm for the black spots of 30 *Rosalia alpina* specimens (ra_01‐ra_30). Each column represents the mean reflectance curve for one individual, calculated from seven black spots (one on the pronotum and six on the elytra).
**Table S3:** Chromatic contrast analysis between random *Rosalia alpina* specimens and the models used for the predation experiment.
**Table S4:** Predation experiment—dataset.

## Data Availability

The data that support the findings of this study are made available as Supporting Information.
